# Correction to “Novel Gene Clusters for Secondary Metabolite Synthesis in Mesophotic Sponge‐Associated Bacteria”

**DOI:** 10.1111/1751-7915.70136

**Published:** 2025-03-26

Chen, N., Liu, L., Wang, J., et al. 2025. “Novel Gene Clusters for Secondary Metabolite Synthesis in Mesophotic Sponge‐Associated Bacteria.” *Microbial Biotechnology* 18, no. 2: e70107. https://doi.org/10.1111/1751‐7915.70107.

In the Acknowledgements and Funding sections, the text ‘41776168’ was incorrect. This should have read as ‘42176101’. Additionally, ‘Ningbo Natural Science Foundation (2021Z04)’ was incorrect. This should have read as ‘Ningbo Key Science and Technology Development Program (2021Z046)’.

The online article has also been updated with these corrections.

We apologise for this error.

